# TNF-α and IFN-γ Participate in Improving the Immunoregulatory Capacity of Mesenchymal Stem/Stromal Cells: Importance of Cell–Cell Contact and Extracellular Vesicles

**DOI:** 10.3390/ijms22179531

**Published:** 2021-09-02

**Authors:** Lucero López-García, Marta E. Castro-Manrreza

**Affiliations:** Immunology and Stem Cells Laboratory, Multidisciplinary Unit of Experimental Research Zaragoza, FES Zaragoza, National Autonomous University of Mexico, Mexico City 09230, Mexico; lucerop624@gmail.com

**Keywords:** mesenchymal stem/stromal cells, immunoregulation, cell–cell contact, extracellular vesicles

## Abstract

Mesenchymal stem/stromal cells (MSCs) have an immunoregulatory capacity and have been used in different clinical protocols requiring control of the immune response. However, variable results have been obtained, mainly due to the effect of the microenvironment on the induction, increase, and maintenance of MSC immunoregulatory mechanisms. In addition, the importance of cell–cell contact for MSCs to efficiently modulate the immune response has recently been highlighted. Because these interactions would be difficult to achieve in the physiological context, the release of extracellular vesicles (EVs) and their participation as intermediaries of communication between MSCs and immune cells becomes relevant. Therefore, this article focuses on analyzing immunoregulatory mechanisms mediated by cell contact, highlighting the importance of intercellular adhesion molecule-1 (ICAM-1) and the participation of EVs. Moreover, the effects of tumor necrosis factor-alpha (TNF-α) and interferon-gamma (IFN-γ), the main cytokines involved in MSC activation, are examined. These cytokines, when used at the appropriate concentrations and times, would promote increases in the expression of immunoregulatory molecules in the cell and allow the acquisition of EVs enriched with these molecules. The establishment of certain in vitro activation guidelines will facilitate the design of conditioning protocols to obtain functional MSCs or EVs in different pathophysiological conditions.

## 1. Mesenchymal Stem/Stromal Cells (MSCs)

MSCs are multipotent stromal cells with the capacity for self-renewal and differentiation. They were originally identified in bone marrow (BM) [1,2] and, currently, cells with similar characteristics can be obtained from different tissues [3,4,5]. Three biological properties have been identified in BM-MSCs: (a) differentiation potential, (b) secretion of trophic factors, and (c) immunoregulatory capacity. These properties have led to their use in different experimental cell therapy protocols designed for the treatment of inflammatory and autoimmune diseases, tissue repair, and regeneration, and to favor the acceptance of transplanted cells, tissues, and organs [3,5,6]. The first clinical trials performed with MSCs bet on their differentiation potential. Currently, it is accepted that the benefit observed in cell therapy protocols is due to the interaction of the three biological properties mentioned above, mainly the association between the secretion of trophic factors and the regulation of the immune response [3]. In particular, this last property is relevant because the regulation of inflammatory processes is essential for tissue regeneration and repair to occur.

## 2. Immunoregulatory Properties of BM-MSCs

**Figure 1:**
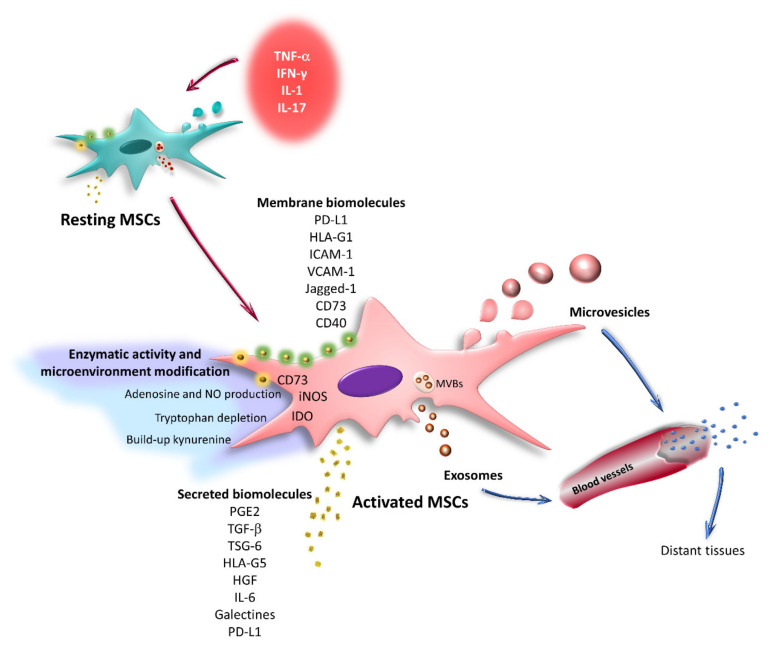
Immunoregulatory mechanisms of activated MSCs. MSCs are generally in a resting state with low or no expression of the molecules involved in their immunoregulatory function. However, when exposed to proinflammatory cytokines such as TNF-α, IFN-γ, IL-1, and IL-17, these cells become activated. This event increases or induces the expression of immunoregulatory molecules in MSCs, which can be secreted or remain attached to the cell membrane. In addition, resting and activated MSCs release extracellular vesicles (exosomes and microvesicles), which are capable of traveling through body fluids and reaching distant sites, where they establish contact with immune cells.

MSCs generate an anti-inflammatory environment by modulating the function of immune cells, such as neutrophils, natural killer (NK) cells, monocytes, macrophages, dendritic cells (DCs), and T and B lymphocytes [7,8,9,10,11,12,13]. It is currently proposed that MSCs modulate the activation, differentiation, and effector function of immune cells by secreting factors through cell–cell contact and extracellular vesicles (Figure 1).

In vitro, preclinical, and clinical studies have documented the importance of an inflammatory environment in the biological properties of MSCs. These cells are generally resting, with low or no expression of the molecules involved in their immunoregulatory function [7]. However, when MSCs are exposed to an inflammatory environment, the expression of these molecules is increased or induced. Therefore, some researchers have suggested that MSCs must be “activated” for efficient immunoregulation. The activation of MSCs is induced by inflammatory cytokines such as tumor necrosis factor-alpha (TNF-α), interferon-gamma (IFN-γ), interleukin (ΙL)-1, and IL-17 [8,9,10,11,12,13,14]. Because TNF-α and IFN-γ are the first cytokines secreted in an inflammatory response, it is relevant to analyze their effects on the expression of molecules involved in the different immunoregulatory mechanisms used by MSCs (Figure 1).

## 3. Immunoregulation Mediated by Secreted Factors

Immunoregulatory mechanisms mediated by secreted factors have likely been the most extensively analyzed to date, and they have been the subject of numerous reviews [5,15]. Therefore, only a general overview of these mechanisms is provided here. The molecules involved in these processes include prostaglandin E2 (PGE2), transforming growth factor-beta (TGF-β), tumor necrosis factor-stimulated gene-6 (TSG-6), hepatocyte growth factor (HGF), human leukocyte antigen-G5 (HLA-G5), IL-6, IL-10, and galectins [5,6,16,17,18]. These mechanisms also include the effects on the microenvironment induced by the intracellular enzymes indoleamine-2,3-dioxygenase (IDO) and inducible nitric oxide synthase (iNOS) [10,19,20], as well as the production of adenosine by the ectonucleotidase CD73 [21] (Figure 1).

MSCs decrease NK cell proliferation, cytotoxicity, and the expression of cytokines through IDO, PGE2, TGF-β, and HLA-G5 [22,23,24,25]. In addition, it has been recently proposed that the secretome of human BM-MSCs is responsible for promoting a regulatory phenotype in macrophages [26] by inducing monocyte differentiation toward an anti-inflammatory M2 phenotype, with a lower secretion of TNF-α and ΙL-12 and an increased secretion of IL-6 and IL-10 [27]. These effects are due to the action of PGE2 secreted by MSCs [28,29,30]. Through similar mechanisms, these cells affect the activation and maturation of DCs and revert mature DCs to an immature state with a lower capacity to present antigens to T lymphocytes [31,32].

MSCs also affect the proliferation and differentiation of T lymphocytes by decreasing the generation of Th1 and Th17 cells and increasing the expansion of regulatory T lymphocytes (Tregs). The main molecules involved in these effects are PGE2, HLA-G5, and IDO [22,31,33,34], the expression of which is increased in MSCs exposed to IFN-γ. Wharton’s jelly-derived MSCs (WJ-MSCs) pretreated with this cytokine are more efficient in inducing the production of Tregs and affect the secretion of IFN-γ, TNF-α, and IL-17 by activated T lymphocytes [35]. Similar results have been obtained with human adipose tissue-derived MSCs (AT-MSCs), in which treatment with IFN-γ increases their immunoregulatory effects on CD4 and CD8 T lymphocytes through mechanisms mainly mediated by IDO [36,37].

In addition, MSCs decrease the proliferation, activation, and maturation of B cells through the secretion of IL-10, TGF-β, PGE2, nitric oxide (ON), and IDO, affecting immunoglobulin production (IgM, IgG, IgA, and IgE) and chemokine receptor expression (CXCR4, CXCR5, and CCR7), which reduces the migration capacity of B cells [38,39,40]. One of the mechanisms used by AT-MSCs to decrease B lymphocyte proliferation is tryptophan depletion mediated by the action of IDO, the expression of which is significantly increased in AT-MSCs exposed to IFN-γ [40]. In turn, murine BM-MSCs stimulated with this cytokine decrease the production of IL-10 by activated B lymphocytes; this effect involves the cyclooxygenase 2 (COX-2) pathway and cell–cell contact [41]. The interconnection of immunoregulatory mechanisms mediated by secreted factors and by cellular contact has also been observed in the generation of Tregs [22,42]. These data demonstrate that MSCs can alter the function of cells of the immune system through paracrine mechanisms, which are linked to mechanisms mediated by cell–cell contact.

## 4. Immunoregulation Mediated by Cell–Cell Contact

Although regulatory factor secretion plays an important role in the immunoregulatory potential of MSCs, several studies have reported the relevance of direct contact with immune system cells for the development of efficient immunoregulation. Molecules expressed in the MSC membrane, such as programmed cell death ligand 1 (PD-L1), human leukocyte antigen-G1 (HLA-G1), CD40, Jagged-1, intercellular adhesion molecule 1 (CD54/ICAM-1), and vascular cell adhesion molecule 1 (VCAM-1), participate in these mechanisms [43,44,45,46,47] (Figure 1). Furthermore, the generation of nanotubes by T lymphocytes to establish contact with MSCs has been reported [48].

**Figure 2:**
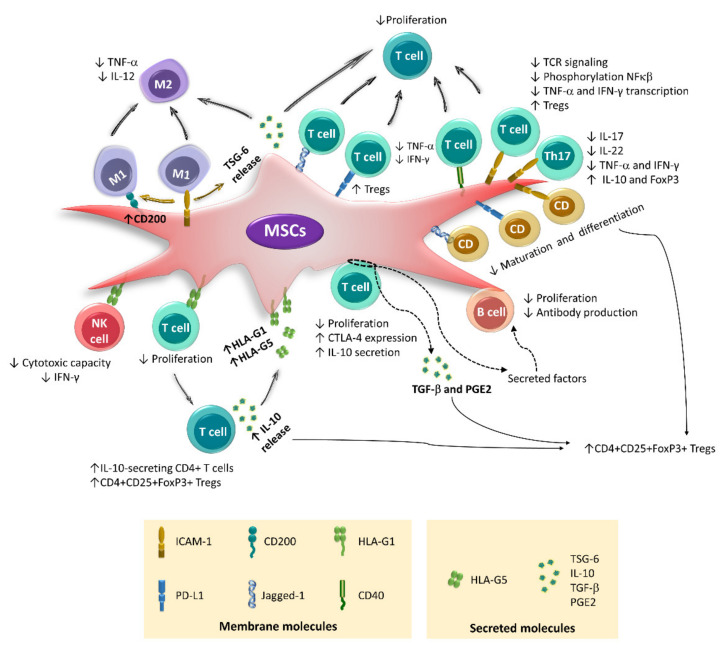
Immunoregulatory mechanisms mediated by cell–cell contact. The main membrane molecules involved in the immunoregulation exerted by MSCs are shown. Cell–cell interactions, in addition to affecting the proliferation, differentiation, and effector function of immune cells, also increase the immunoregulatory capacity of MSCs. The contact of M1 macrophages with MSCs through ICAM-1 induces an M2 phenotype in macrophages, while in MSCs, the expression of CD200 and TSG-6 is increased (brown arrows), which also favors the differentiation of M2 macrophages. Conversely, ICAM-1, PD-L1, and jagged-1 decrease the secretion of cytokines and proliferation of activated T lymphocytes, as well as the maturation and differentiation of DCs. Furthermore, CD40 affects the proliferation of T cells, while HLA-G1 induces Tregs differentiation. HLA-G1 is also involved in the decrease in effector function of NK cells. Conversely, direct contact of MSCs with T lymphocytes (circle with dotted lines) induces changes in T lymphocytes and stimulates the secretion of TGFβ and PGE2, as well as factors that affect the function of B lymphocytes (dotted lines). However, it is unknown exactly which molecules are involved in this interaction.

Using transwell systems, direct cellular contact has been identified as an essential event for placenta-derived MSCs (PL-MSCs), which express HLA-G1 upon stimulation with IFN-γ to decrease the cytotoxicity of NK cells toward the K562 tumor line [32,36,37]. In addition, the participation of HLA-G1 and HLA-G5 in decreasing the proliferation of alloantigen-activated peripheral blood mononuclear cells (PBMCs) and differentiation of CD4+CD25+Forkhead box P3 (FoxP3)+ Tregs has been described. HLA-G1 is an isoform that remains bound to the MSC membrane, while HLA-G5 is secreted [22,49]; the latter can stimulate IL-10 production by activated T lymphocytes. IL-10 stimulates the expression of both isoforms in MSCs, generating a positive feedback mechanism [22,50]. HLA-G1 is likely the first molecule involved in the establishment of this interaction. This hypothesis is supported by a study in which basal levels of HLA-G1 mRNA were detected in MSCs derived from the BM, AT, and fetal liver. Moreover, overexpression of this isoform in AT-MSCs increases their immunoregulatory capacity toward activated T lymphocytes [44]. Likewise, cell–cell contact between MSCs and populations enriched in CD3+ T lymphocytes is essential for the increase in IL-10 levels detected in the supernatants of these cocultures [51,52]. This process is important because IL-10 participates in the generation of Tregs by MSCs and increases the expression of programmed cell death protein 1 (PD-1) in CD4+CD25+ cells, which is associated with greater immunoregulatory activity [53] (Figure 2).

In BM-MSC cocultures with enriched CD4+ lymphocyte populations, the generation of CD4+CD25+FoxP3+ lymphocytes involves the participation of TGF-β and PGE2, but direct contact between the two cell types is an indispensable prerequisite [42]. Similar observations have been made in co-cultures with tonsil-derived MSCs (T-MSCs), where cell contact is essential to decrease the proliferation of CD4 T lymphocytes, as well as the differentiation of CD4+TNF-α+ and CD4+IFN-γ+ cells [47]. Likewise, our working group showed that the cell–cell interaction between activated CD3+ T lymphocytes and MSCs derived from BM or umbilical cord blood (UCB) is necessary to increase the expression of cytotoxic T lymphocyte-associated protein 4 (CTLA4), a molecule that is constitutively expressed by Tregs [51]. Interestingly, the direct contact between MSCs and CD3+ T lymphocytes, in addition to affecting the function of these same T lymphocytes, can also affect B lymphocytes.

Direct contact of human BM-MSCs with CD3+ T lymphocytes apparently inhibits the proliferation, differentiation, and production of antibodies by B cells because these effects are observed in cocultures of MSCs with CpG-activated peripheral blood lymphocytes but not in cocultures with sorted B cells. Moreover, the use of transwells in the first system reverses the inhibitory effects, while the addition of sorted T cells to the second system recovers the immunoregulatory activity of MSCs. The authors propose that the immunosuppressive effect of MSCs on B cells is mediated by soluble factors secreted during the cell–cell interaction of MSCs with CD3 T lymphocytes [54]. However, other studies have observed that direct contact between MCSs and populations enriched in CD19+ B cells affects the functions of the latter (Figure 2). In this regard, it has been documented that resting human AT-MSCs do not decrease the proliferation of B lymphocytes but favor the differentiation of regulatory CD19+CD24hiCD38hi B lymphocytes and increase the expression of IL-10. In turn, AT-MSCs exposed to IFN-γ do not favor regulatory B lymphocyte differentiation but reduce B lymphocyte proliferation and inhibit IgG production. These effects are more efficient when direct contact between MSCs and CD19+ B cells is allowed [40].

The current evidence highlights the importance of cell–cell contact in the immunoregulatory activity of MSCs by favoring the development of mechanisms mediated by soluble factors. Therefore, we must improve our knowledge of these interactions and understand the participation of the different membrane-bound molecules. In this sense, several recent reports have indicated the relevance of adhesion molecules in the interaction of MSCs with immune cells, particularly intercellular adhesion molecule-1 (ICAM-1) [19,55,56].

ICAM-1 is a highly glycosylated protein that belongs to the immunoglobulin superfamily of cell adhesion molecules. It is expressed on fibroblasts, endothelial cells, antigen-presenting cells, and lymphocytes and participates in cell–cell and cell–matrix adhesion, modulating cell migration processes [57]. This adhesion molecule is a key regulator of the immune response, is involved in the differentiation of monocytes and DCs, and participates in the immunological synapse between antigen-presenting cells and T lymphocytes, which modulates the activation and differentiation of the latter [58,59]. It has been observed that ICAM-1 promotes a decrease in the differentiation of Th17 lymphocytes [59] and the establishment of memory CD8 T lymphocytes [60,61]. In addition, endothelial cells exposed to TNF-α show increased expression of this adhesion molecule, which is important in inducing and regulating the immune response, since the contact of DCs with lymphatic endothelial ICAM-1^hi^ cells decreases the expression of CD86 and the capacity of DCs to induce the activation and proliferation of T lymphocytes [62]. Similar mechanisms have been identified in MSCs.

Resting MSCs express ICAM-1 at low levels; however, their expression is increased and induced in the presence of an inflammatory environment, which favors its interaction with immune cells [19,45,63]. ICAM-1 promotes the adhesion of murine BM-MSCs to DCs, inhibiting the maturation and differentiation of the latter [63]. Additionally, during exacerbated inflammatory responses, ICAM-1 favors the interplay of human BM-MSCs with M1 macrophages, which induces the generation of M2 anti-inflammatory macrophages [64]. ICAM-1 polarization has been observed in cell contact areas, which results in the formation of an “unconventional synapse” capable of modulating the function of both cells [64]. Likewise, direct contact between M1 macrophages and murine BM-MSCs increases the immunoregulatory activity of these cells toward the same macrophages and the proliferation of CD4+ T lymphocytes. In addition, the interaction of MSCs with M1 macrophages in a contact-dependent or contact-independent manner increases the expression of CD200 in MSCs, another membrane molecule involved in the transition from M1 to M2 [65] (Figure 2).

Human MSCs derived from BM [66], umbilical cord (UC), and TA [67] more efficiently inhibit PBMC proliferation when direct contact occurs between the two cell types. In cocultures of CD3+ cells activated in the presence of MSCs derived from BM, amnion, or UCB [12,51,52,68,69], the inhibition of cell contact practically reestablishes the proliferation of T lymphocytes. Additionally, the generation of regulatory T cell populations by MSCs involves the participation of several membrane molecules, including HLA-G1, PD-L1, VCAM-1, and ICAM-1 [19,22,70] (Figure 2).

The ICAM-1 blockade restores the proliferation of activated T lymphocytes in the presence of BM-MSCs [55] or AT-MSCs [56] and inhibits the generation of FoxP3+ cells [56]. In addition, it has been proposed that the direct contact of human BM-MSCs with T lymphocytes through ICAM-1 and CD43 is a critical event in the immunoregulatory activity of these cells. This interaction is capable of immediately decreasing the transcription of TNF-α and IFN-γ in activated T lymphocytes because ICAM-1 expressed on MSCs regulates T cell receptor (TCR) signaling [71]. Moreover, this adhesion molecule participates in the interplay of human BM-MSCs with Th17 cells through a mechanism that involves the interaction of CCR6 with its ligand CCL20 and the induction of a conformational change in CD11a/CD18 that promotes its binding to ICAM-1. This event increases when MSCs are exposed to TNF-α and IFN-γ due to the increased expression of ICAM-1. Subsequently, the production of IL-17, IL-22, IFN-γ, and TNF-α by differentiated Th17 cells is decreased, and a regulatory phenotype with increased expression of IL-10 and FoxP3 is induced [34] (Figure 2). Importantly, the effects of MSCs on differentiated Th17 cells decrease when a transwell is used, but they are not completely inhibited, since PGE2 is also involved in these processes.

The importance of ICAM-1 in the immunoregulatory effects exerted by MSCs has also been observed in preclinical studies. In a murine model of graft-versus-host disease (GVHD), an infusion of ICAM-1+ BM-MSCs reduced the expression of inflammatory cytokines and the percentage of Th1 lymphocytes. Additionally, it increased the migratory capacity of MSCs and the differentiation of T cells toward a regulatory phenotype associated with immune tolerance [63]. Likewise, in a murine model of inflammatory bowel disease, the administration of BM-MSCs overexpressing ICAM-1 decreased the percentage of Th1 and Th17 cells, as well as the transcription of IFN-γ and IL-17. In addition, these cells increased the differentiation of Tregs and the transcription of FoxP3, all of which are associated with a decrease in lesions [45].

On the other hand, the expression of jagged-1, a Notch ligand, on the surface of MSCs has been implicated in its immunoregulatory activity toward DCs and T lymphocytes. Blocking jagged-1 with antibodies has been observed to decrease the inhibitory effect of human BM-MSCs on CD4+ lymphocyte proliferation [46]. In murine BM-MSCs, the participation of jagged-1 in the expansion of CD4+CD25+FoxP3+ Treg cells and the induction of DCs with a semimature phenotype has been confirmed, which also favors the differentiation of Tregs from CD4+CD25-FoxP3- populations [72] (Figure 2).

Another important molecule involved in contact-mediated immunoregulation is PD-L1, the expression of which increases in MSCs treated with IFN-γ [9,67,73,74,75,76]. This molecule contributes to the decrease in the differentiation and maturation of DCs [73]. In addition, it affects the activation, proliferation, and effector function of T lymphocytes [67,74,77,78], increasing the generation of Treg cells [73] and decreasing the secretion of TNF-α and IFN-γ in activated T lymphocytes [79]. A recent study shows that this last effect is also mediated by CD40, whose expression increases in T-MSCs activated with TNF-α and IFN-γ [47] (Figure 2). It is important to mention that, although PD-L1 is a molecule present in the membrane of MSCs, some studies have suggested that it can also be secreted and that this form is capable of decreasing the expression of CD25 and IL-2 in activated T lymphocytes [78]. Interestingly, this working group found that PD-L1 is secreted freely or bound to extracellular vesicles, structures that can mediate the immunoregulatory effect of MSCs.

## 5. Immunoregulation Mediated by Extracellular Vesicles

Given the importance of cellular contact in the immunoregulatory activity of MSCs, it is not clear how this event might occur in the physiological context, since a poor grafting capacity and low viability of the transplanted cells have been observed in patients over the course of days [80]. Thus, it is currently proposed that MSCs release extracellular vesicles (EVs), structures that have been recognized as an important mechanism of paracrine and endocrine cellular communication [81,82], to mediate the immunoregulatory effects of MSCs even at distant sites.

**Figure 3:**
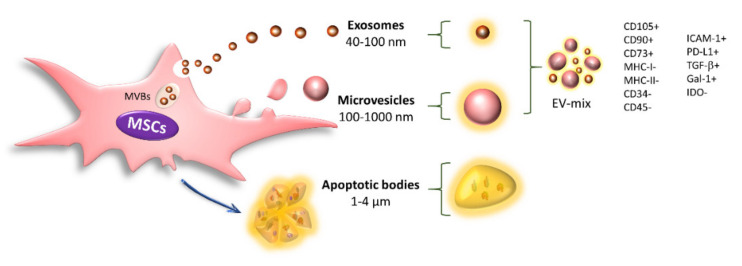
Classification of extracellular vesicles. EVs are classified into exosomes, microvesicles, and apoptotic bodies. Exosomes and MVs are important intercellular communication mechanisms. These structures transport different molecules inside or on the membrane, through which they modify the behavior of their target cells. Some of the main molecules identified in EVs released by resting MSCs are shown.

EVs are mainly classified based on their biogenesis, size, and shape into exosomes, microvesicles, and apoptotic bodies. Exosomes are homogeneous vesicles with a size ranging from 40 to 100 nm that are derived from multivesicular bodies (MVBs) and secreted through fusion of the MVBs with the cell membrane. In addition, they are positive for CD63, CD9, CD81, and TSG101. Microvesicles (MVs) are a heterogeneous population ranging from 100 to 1000 nm in size that originate from direct protrusions of the cell membrane that detach from the surface, and these structures retain many characteristics of their cells of origin [83,84]. Finally, apoptotic bodies are EVs with a diameter of 1000–4000 nm that are released from the plasma membrane when the cells undergo apoptosis. These structures are characterized by the presence of phosphatidylserine in the outer membrane and contain organelles, histones, and fragmented DNA [48,83,84] (Figure 3).

All cells of an organism release exosomes and MVs, which are an important mechanism of intercellular communication because they can reach the bloodstream and travel to distant sites where they establish contact with their target cells and influence their biological behaviors. Through proteomics, genomics, lipidomics, and metabolomics assays, exosomes and MVs have been shown to contain proteins (ligands, receptors, adhesion molecules, enzymes, cytokines, and growth factors), lipids, metabolites, mRNAs, and microRNAs, through which they modulate their target cells [83,84,85,86,87]. Currently, there is still controversy regarding the nomenclature and methods of obtaining these structures. The terms exosome, MV, and EVs are used as synonyms in several reports, which may confuse the interpretation of the results. These discrepancies highlight the importance of characterizing and analyzing the specific function of each type of EV [88,89]. Therefore, in each article cited in this review, we specify whether the study was performed with exosomes, MVs, or a mixture of exosomes and microvesicles (EV-mix) based on an analysis of the method used to obtain them (Table 1).

Resting MSCs release exosomes and MVs with positivity for the characteristic markers of these cells (CD105, CD90, and CD73) and the absence of MHC-I, MHC-II, CD34, and CD45 [48,90,91,92,93,94,122,123]; they also transport immunoregulatory molecules such as PD-L1, Gal-1, and TGF-β [90,124] (Figure 3).

Currently, EV-mediated communication is proposed to involve the direct contact of these structures with their target cells. In this regard, CD44 participates in the uptake of the EV-mix by bone marrow-derived mast cells [95] and human alveolar epithelial type II cells [96]. Likewise, phosphatidylserine transported on the surface of exosomes released by human BM-MSCs facilitates the uptake of these structures by human umbilical vein endothelial cells (HUVECs) [97]. Furthermore, it has been shown that these structures can be captured by granulocytes, NK cells, mast cells, monocytes, CDs, and T and B lymphocytes [87,93,95,98,99,125], which affects the function of these immune cells.

The EV-mix released by resting human BM-MSCs induces an M2 phenotypic switch in monocyte-derived macrophages, along with a higher expression of CD206 and PD-L1 [100]. Similar observations have been made with exosomes released by MSCs from breast tumors, which promote the differentiation of myeloid cells into M2-type immunosuppressive macrophages with high expression levels of CD206, PD-L1, and IL-10 and higher L-arginase activity [101]. In contrast, a recent study reports that exosomes and MVs released by murine TA-MSCs are unable to decrease the secretion of pro-inflammatory cytokines (IL-1β y TNF-α) by peritoneal macrophages stimulated with LPS [112], while the EV-mix released by resting human BM-MSCs decreases the uptake of antigens by immature DCs, suggesting that these structures potentially affect this key event in the maturation of DCs. Additionally, mature DCs exposed to these structures show reduced CD83, CD38, and CD80 expression, as well as IL-6 and IL-12p70 secretion, but increased TGF-β production [87]. It is still necessary to determine whether the changes in the differentiation of macrophages and DCs induced by EVs affect the ability of these cells to induce the activation, proliferation, and differentiation of T lymphocytes.

Currently, the results regarding the effect of EVs on T lymphocytes are controversial. In vitro tests have shown that the EV-mix released by murine and human BM-MSCs can increase the generation of the Tregs CD4+CD25+FoxP3+ and CD4+CD25+CD127^low^FoxP3+, respectively [90,102]. They also decrease the proliferation of PBMCs and stimulate the expression of IL-10 and TGF-β [90]. Likewise, the EV-mix released by murine AT-MSCs affects the proliferation of T lymphocytes and the generation of Th1 cells. In addition, they promote the differentiation of a population of FoxP3+ IFN-γ+ cells, which are capable of decreasing the proliferation of CD4 and CD8 T lymphocytes [94]. Moreover, exosomes released by human BM-MSCs and EV-mix derived from AT-MSCs reduce the proliferation of these cells [37,92,99] and the secretion of IFN-γ and IL-17 while increasing the production of IL-10, IL-6, and PGE2, as well as the differentiation of Tregs [92]. In contrast, Matula et al. (2017) observed that exosomes and MVs (fractions analyzed separately) released by human AT-MSCs are incapable of altering the proliferation of T lymphocytes and the secretion of IFN-γ [48]. A similar result was obtained with the EV-mix released by human BM-MSCs [98], and it has been proposed that these structures are not taken up by CD3+ T cells [87]. However, interestingly, it has been observed that the exosomes and MVs released by activated T lymphocytes can be taken up by MSCs and increase the secretion of PGE2 [48]. This evidence indicates that EVs are also involved in the feedback mechanisms established between MSCs and immune cells. The above findings highlight the importance of further studies of these structures.

Studies conducted with an EV-mix [125] or exosomes [99] released by human BM-MSCs have shown that these structures also exert immunosuppressive effects on B lymphocytes, since they decrease their proliferation and differentiation, significantly affecting the production of IgM, IgA, and IgG [99,125].

Furthermore, the immunoregulatory capacity of EV-MSCs has been observed in preclinical models. In animal models, the EV-mix constitutively secreted by UCB- and WJ-MSCs attenuated the kidney damage caused by ischemia [85,91], as well as the clinical manifestations in the skin of mice with chronic GVHD [103]. These effects are related to reduced infiltration and activation of macrophages, an increase in IL-10 levels, and a decrease in TNF-α levels in damaged tissues [91,103]. Similar results have been obtained in a murine model of pulmonary ischemia, where the administration of an EV-mix released by WJ-MSCs decreased tissue damage, which was related to lower concentrations of IL-17 and TNF-α, as well as an increase in the levels of PGE2, IL-10, and the keratinocyte growth factor in the bronchoalveolar fluid [104]. Moreover, in a mouse model of acute GVHD, the administration of an EV-mix released by human BM-MSCs stimulated Treg differentiation [102].

In addition, the administration of EV-mix released by resting UC-MSCs, in a mouse liver ischemia/reperfusion injury model, significantly attenuated liver tissue damage, with a decrease in the percentage of intrahepatic CD4+CD154+ and CD68+ cells, and less production of TNF-α and IFN-γ [113]. A similar anti-inflammatory effect has been observed with EV-mix administered in a rat model of collagen-induced arthritis. Attenuation of the severity of the disease is observed, which is associated with less proliferation and increased apoptosis of splenic T cells. In addition, the differentiation of CD4+IL-17+ cells is affected and the differentiation of T reg CD4+CD25+FoxP3+ is favored. All of the above is accompanied by lower serum levels of IL-17 and an increase in IL-10 and TGF-β [114]. Similarly, the administration of exosomes released by TA- or PL-MSCs in an induced colitis mouse model reduces local and systemic inflammation. In the colon tissue, a lower expression of IL-1β, IL-6, TNF-α, IFN-γ, IL-17, and IL-12 is observed, as well as an increase in IL-10 and TFG-β [115,116]. Moreover, the levels of reactive oxygen species (ROS), the expression of apoptotic proteins, and metalloproteinase (MMP-2 and MMP-9) are reduced [116].

Currently, only one clinical trial conducted with EVs released by resting MSC has reported results. The administration of two doses of EV-mix obtained from UCB-MSCs to patients with grade III-IV chronic kidney damage induced an improvement in renal function, decreasing inflammation and TNF-α levels, and increasing plasma levels of TGF-β and IL-10 [105]. In addition, an increase in the percentage of PD-L1+ exosomes was detected in the plasma of patients with acute GVHD treated with clinical-grade WJ-MSCs, suggesting that these structures are potential mediators of the therapeutic effect [106]. This evidence indicates that the EVs released by MSCs may exert an immunosuppressive effect similar to that observed for the cells. EVs have the advantage that their content and possible therapeutic effect are not affected by the microenvironment, which is a disadvantage associated with the use of whole cells. However, it is necessary to carry out clinical trials to determine this, in this regard, there are currently 13 clinical trials registered on the clinicaltrials.gov page, in which the use of EVs released by resting MSCs in the treatment of different pathologies is proposed.

## 6. Effect of the Inflammatory Microenvironment on the Immunoregulatory Capacity of MSCs

As already mentioned, the inflammatory environment modulates the immunoregulatory capacity of MSCs, and this phenomenon has been reported in preclinical and clinical studies. These cells exert a greater therapeutic effect when administered to patients at intermediate stages of the disease due to the presence of an inflammatory environment that stimulates the immunoregulatory properties of MSCs. High serum IFN-γ levels may be a favorable prognostic marker to predict the therapeutic success of UCB-MSCs in patients with lupus erythematosus [126]. Similar observations have been made in the treatment of GVHD, where the administration of MSCs significantly decreased the symptoms of the disease in patients with an acute GVHD refractory to steroids [3,127]. In addition, exposure of AT-MSCs to synovial fluid from patients with rheumatoid arthritis was recently shown to induce overexpression of COX-2, IDO, IL-6, TSG-6, ICAM-1, VCAM-1, and PD-L1 mRNA; using antibodies, the authors determined that these changes were mainly induced by the action of TNF-α [128]. Based on these findings, numerous laboratories have focused on analyzing the effect of proinflammatory cytokines on the different biological properties of MSCs to establish in vitro conditioning protocols that improve the immunoregulatory potential of these cells and, therefore, their therapeutic effect. It is important to mention that currently all clinical trials have used resting MSCs and only one study registered in clinicaltrials.gov will use IFN-γ-primed MSCs in adult and pediatric patients undergoing hematopoietic cell transplantation for the treatment of acute leukemia and myelodysplastic syndrome (NCT04328714). However, the therapeutic effect of MSCs stimulated with pro-inflammatory cytokines has been analyzed in various preclinical models.

In a murine model of dextran sulfate-induced colitis, the administration of BM-MSCs treated with IFN-γ decreased mucosal damage and improved survival rates. These effects were associated with increased migratory and immunoregulatory capacities of MSCs since they were more efficient at inhibiting the inflammatory response mediated by Th1 cells [129]. Likewise, the administration of human BM-MSCs treated with IFN-γ decreased tubular injury and improved renal function in a murine model of acute renal injury with cisplatin, and the changes were associated with an increased secretion of IL-10 by activated MSCs [130]. Similar results have been obtained using mice with GVHD, in which the signs of the disease were prevented and their survival increased [131]. In addition, the administration of human BM-MSCs activated with IFN-γ promotes bone regeneration in mice with calvarial lesions. These positive effects were mainly due to an increase in the expression of IDO by human BM-MSCs [10], which affects T lymphocyte proliferation in vitro [132].

A conditioned medium from rat BM-MSCs exposed to TNF-α, IL-1β, and NO (cytokines released by irradiated epithelial cells) was administered to rats with radiation-induced intestinal damage, resulting in decreased structural and functional damage in the intestine and increased survival. MSCs exposed to this inflammatory environment showed an increased capacity to decrease apoptosis, stimulate the proliferation of intestinal epithelial cells, and generate intestinal stem cells. Additionally, they reduced IL-1β, IL-6, and TNF-α levels and increased the secretion of IL-10 and the differentiation of CD4+FoxP3+ cells, which together decreased the local and systemic inflammatory response in the animals [133]. Similarly, exposure of AT-MSCs to the plasma of patients with acute GVHD increased their ability to induce Treg differentiation in vitro [134]. These results reveal the importance of the design of in vitro conditioning strategies that ensure the therapeutic success of these cells under different pathophysiological conditions. Recently, several studies have focused on analyzing the effect of IFN-γ on MSCs; however, the importance of TNF-α as the first stimulus has recently been highlighted.

## 7. Effects of TNF-α and IFN-γ on the Expression of Immunoregulatory Molecules by MSCs

TNF-α is a pleiotropic cytokine that functions in an autocrine, paracrine, or systemic manner; is expressed mainly by macrophages, DCs, and lymphocytes; and is involved in numerous inflammatory, immunomodulatory, and tissue regeneration pathways. Alterations in its secretion or activity are associated with inflammatory and autoimmune diseases [135]. During an inflammatory process, TNF-α is one of the first cytokines secreted by immune system cells and can increase (prime) or decrease (desensitize or tolerate) the ability of cells to respond to other stimuli [135,136,137]. Nevertheless, there are few reports on the effect of TNF-α on MSCs. Some studies indicate that this cytokine does not alter the expression of immunoregulatory molecules in these cells. In contrast, TNF-α has been proposed to provide the initial stimulus for MSC priming, since it is the first cytokine released by activated T lymphocytes [30,107,138].

**Figure 4:**
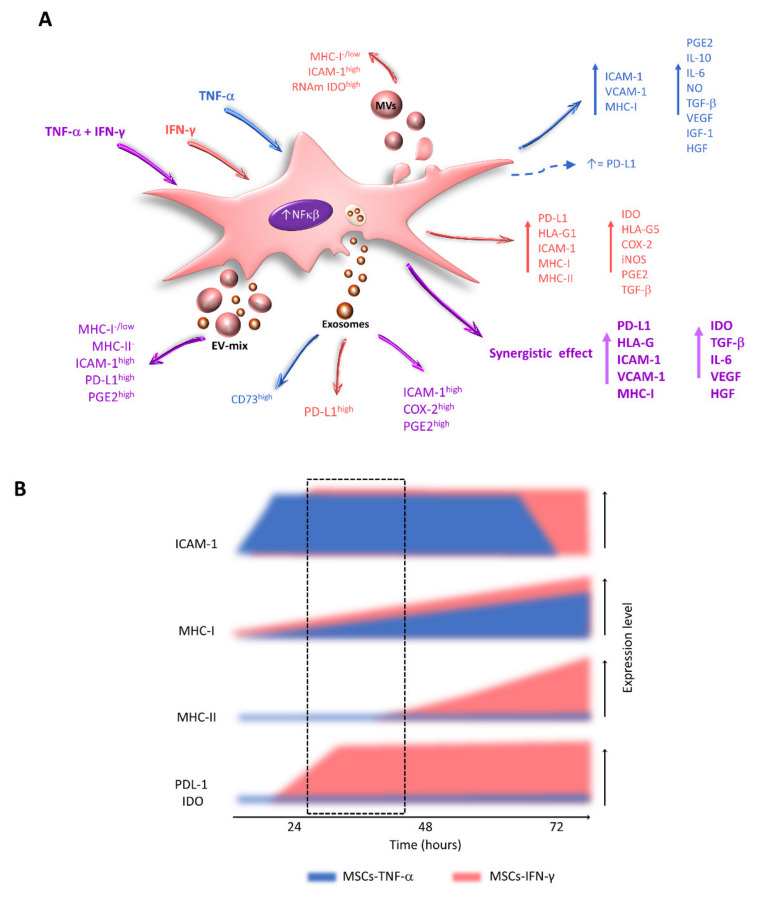
Effect of TNF-α and IFN-γ on the expression of immunoregulatory molecules by MSCs. (**A**) Changes in the expression of immunoregulatory molecules observed in MSCs stimulated with TNF-α (blue), IFN-γ (red), or TNF-α and IFN-γ (purple) are indicated. The changes in exosome load, MVs, and EV-mix released by MSCs treated with the indicated cytokines are also shown. (**B**) Temporal effect of TNF-α and IFN-γ on the expression of immunoregulatory and immunogenic molecules by MSCs. Between 24 and 48 h of treatment with TNF-α, MSCs increase the expression of ICAM-1 but not MHC-I and MHC-II. Simultaneously, IFN-γ stimulates the expression of ICAM-1, PD-L1, and IDO but not MHC-II. These events generate a window in which MSCs with high immunoregulatory capacity and low immunogenic potential can be obtained (dotted-line box).

Treatment of BM-MSCs with TNF-α does not affect the expression of IDO [14] but increases the levels of PGE2, IL-10, IL-6, NO [14,31,139], and ICAM-1 [34,140]. In particular, in human BM-MSCs, TNF-α induces the expression of ICAM-1 after 6 h of exposure, reaching the highest levels at 24 and 48 h [107]; this event is associated with a greater migratory capacity [140]. Likewise, at 24 h, treatment with TNF-α increases the levels of VCAM-1 [141], vascular endothelial growth factor (VEGF), insulin-like growth factor 1 (IGF-1), and HGF [142,143], while at 48 h, significantly higher levels of IL-6 are detected [16]. In addition, in rat UCB-MSCs, TNF-α stimulates the expression of TGF-β and IL-10 [144]. Thus, TNF-α favors the immunoregulatory activity of MSCs and, given the importance of the mechanisms that involve cell contact, it is important for this cytokine to immediately increase the expression of adhesion molecules (Figure 4).

Moreover, it has been reported that TNF-α does not affect the expression of PD-L1 [14] or that it increases its expression but to lower levels than those observed in MSCs stimulated with IFN-γ [75,78]. These contradictory results highlight the importance of further research on the effects of this cytokine on MSCs (Figure 4).

IFN-γ is a key cytokine involved in the induction of innate and adaptive immune responses. It is produced mainly by Th1 lymphocytes, CD8, and NK cells [145]. This cytokine stimulates or increases the expression of major histocompatibility complex (MHC) molecules on the surface of various cell types, including MSCs [25,67,146]. It also induces the expression of IDO and PD-L1 [10,12,18,36,73,74,78,147,148] and increases the expression of HLA-G1, HLA-G5, ICAM-1 [34,43,149], COX-2, and iNOS [10,19,20], as well as the secretion of PGE2 [14,31] and TGF-β [18,25]. These events translate into a greater immunoregulatory capacity of MSCs. Numerous reports have described the effects of IFN-γ, which, while providing an important stimulus, is not the only molecule to which MSCs are exposed, indicating the need to explore the effects of other cytokines (Figure 4).

## 8. Effects of the Combination of TNF-α and IFN-γ on the Immunoregulatory Capacity of MSCs

Few studies have analyzed the effect of combined cytokines on the biology of MSCs. Doing so would be relevant since, in the physiological context, the activation of these cells would occur in response to the presence of different stimuli, the balance of which would dictate the MSC phenotype and function. In support of these findings, some studies have determined that MSCs treated with mixtures of proinflammatory cytokines have a greater immunoregulatory capacity [30,102,150].

In vitro studies have shown that TNF-α and IFN-γ exert a powerful synergistic effect on the expression of immunoregulatory molecules in BM-MSCs [75,107,150], including TGF-β, IL-6, VEGF [150,151], HGF [14], IDO, PD-L1, and HLA-G [75,78,150], as well as ICAM-1, VCAM-1 [55,107], and IL-6 [150]. Similar results have been obtained with UC-MSCs [152] (Figure 4). In addition, a proteomics study carried out with BM-MSCs stimulated with TNF-α and IFN-γ reported a higher expression of IL-4, IL-10, IL-12, IL-15, PD-L1, IDO, and HLA-G, as well as the chemokines CCL5, CXCL9, CXCL10, and CXCL11 [75]. The researchers proposed that the combined treatment eliminated the variation in the expression of cytokines and chemokines intrinsic to each donor [75]. Nevertheless, to date, no study has analyzed whether the synergistic effect of TNF-α and IFN-γ on the expression of immunoregulatory molecules translates into a greater immunoregulatory capacity of MSCs compared to these same cells activated with IFN-γ alone. To date, only one study has reported that human BM-MSC spheroids stimulated with this cytokine mixture could more efficiently decrease the production of TNF-α by macrophages than MSCs stimulated with the same cytokine [150].

Other studies have analyzed the effect of TNF-α and IFN-γ on MSCs. However, due to the experimental design of these works, it is not possible to confirm the presence of a synergistic effect. In this regard, it has been observed that the stimulation of TA-MSCs with both cytokines increases the expression of immunoregulatory molecules, such as IDO, PGE2, IL-10, IL-6, IL-18, CCL-2 [108], TSG-6 [153], and HLA-G5 [154]. Likewise, in BM-MSCs, they increase the expression of TGF-β [155], ICAM-1 and VCAM-1 [102,156], IDO, TSG-6 [157,158], PD-L1 [102], HLA-G5, and factor H, a primary complement inhibitor [159].

It has been determined that human BM-MSCs activated with TNF-α and IFN-γ increase the expression of IDO, which induces the conversion of monocytes to IL-10-secreting CD206+ M2 immunosuppressive macrophages; these features contribute to the immunoregulatory effect of MSCs on T lymphocyte proliferation [157]. BM-MSCs and TA-MSCs treated with both cytokines also directly decrease the proliferation of T lymphocytes [154]. The few studies conducted to date highlight the need to determine whether the synergistic effect induced by TNF-α and IFN-γ on the expression of immunoregulatory molecules truly increases the immunoregulatory activity of these cells, which would indicate a need to use TNF-α in conditioning protocols.

On the other hand, the effect of other cytokine combinations has recently been analyzed, and a synergistic effect of TNF-α and IL-10 on the secretion of PGE2 by BM-MSCs has been observed [30]. In human UCB-MSCs, a synergistic effect of IFN-γ and IL-1β on the expression of PGE2 and IDO has been reported, which increases their ability to decrease T lymphocyte proliferation and Th1 differentiation; in addition, they are more efficient in Treg induction [33].

## 9. Important Aspects to Consider for In Vitro Activation Protocols

The exposure of MSCs to a cocktail of cytokines that increases their immunoregulatory capacity is a challenge that must consider two main factors: the temporal effects of the cytokines and their concentration. The balance between these factors will affect the viability, proliferation, immunogenic potential, and expression of immunoregulatory molecules. In addition, given the importance of contact mechanisms, favoring the presence of these molecules in the membrane of cells and extracellular vesicles is indispensable.

## 10. Alterations in Cell Morphology and Proliferation

Numerous studies have reported adverse effects when using high concentrations of IFN-γ for prolonged periods, including cell cycle arrest, decreased proliferation [9,146,160,161], and changes in the morphology of MSCs. In particular, an increased cell size is associated with the senescence of cultured cells [162,163] and a lower immunoregulatory capacity [164].

Stimulation of human BM-MSCs with high concentrations of IFN-γ for 7 and 14 days increases the senescence of these cells, resulting in a lower clonogenic capacity and an increasing trend in the percentage of apoptotic cells [165]. Likewise, treatment of UCB-MSCs with TNF-α (20 ng/mL for 48 h) induces apoptosis, and this effect is more evident in the presence of IFN-γ (50 ng/mL) [166]. Similar observations have been reported in human AT-MSCs stimulated with TNF-α and IFN-γ for 48 h, which show changes in morphology and decreased proliferation in a dose-dependent manner [108]. These changes have also been detected in BM- and infrapatellar fat pad-derived MSCs exposed to these cytokines [156].

Adverse effects on morphology and decreased proliferation have also been described in TA-MSCs treated with 50 ng/mL IFN-γ, 20 ng/mL TNF-α, and 10 ng/mL IL-6 for 7 days [160]. MSCs derived from gingival tissue (G-MSCs) exposed to IL-1β (1 ng/mL), TNF-α (10 ng/mL), and IFN-γ (100 ng/mL) for 72 h practically lose their clonogenic capacity after 14 days of exposure to this mixture of proinflammatory cytokines [167]. All these changes associated with the induction of senescence in cultures should be avoided in conditioning strategies. This is because the use of MSCs in therapeutic protocols requires an ex vivo expansion process to obtain the necessary number of cells, and which generally induces replicative senescence. Proliferation arrest decreased adipogenic, osteogenic, and chondrogenic potential for differentiation, as well as decreased immunoregulatory capacity, have been observed in the late-passage of human-BM-MSCs [168,169]. Similar results have been seen in MSCs whose senescence is induced by radiation [170], oxidative stress [171], or exposure to pro-inflammatory cytokines [172]. Because such alterations can affect the therapeutic efficacy of these cells, it has currently been proposed to remove senescent cells before being administered to patients [173] or to establish non-senescent MSC lines [120].

Senescent cells have been shown to possess a characteristic secretome, called senescence-associated secretory phenotype (SASP) [174], characterized by the production of cytokines, chemokines, growth factors, proteases, and reactive oxygen species; these together modify the local and systemic environment. The SASP acts in an autocrine way, reinforcing the arrest of cell growth, while in a paracrine way it induces senescence in neighboring cells, remodels the extracellular matrix, affects the stem cell niches, and stimulates the recruitment and activation of immune cells favoring an inflammatory state [174,175,176]. It has been reported that TA-MSC treatment of healthy donors with 20 ng/mL of TNF-α for 24 h induces SASP with increased expression of IL-6, IL-8, and monocyte chemotactic protein-1 (MCP-1) [172]. Even UC-MSCs treated with 5 ng/mL of TNF-α for three days have been used as a model of senescence [177]. This indicates the need to analyze in detail the effect of cytokine concentrations and exposure times on MSC functions. This will avoid increasing cellular senescence and inducing alterations during conditioning protocols.

Interestingly, the exposure of AT-MSCs to an inflammatory environment generated by activated T lymphocytes in a mixed culture does not affect their proliferation or morphology [160]. This result highlights the importance of using concentrations closer to those identified in different pathophysiological contexts in conditioning protocols. It is important to note that TNF-α and IFN-γ concentrations in the serum or plasma of patients with inflammatory diseases are found at the nanogram or picogram level [134,178,179,180,181]. In fact, the exposure of AT-MSCs to the plasma of patients with GVHD has recently been proposed as an in vitro conditioning strategy [134].

## 11. Increased Immunogenicity

The increase in the immunogenic capacity of MSCs subjected to conditioning protocols is an undesired characteristic. In the basal state, these cells express low levels of MHC-I and do not express MHC-II [51,67,74,107,182]. However, the expression of these molecules increases significantly when MSCs are exposed to proinflammatory cytokines such as TNF-α, IFN-γ, and IL-17 [9,148,183], which increase their immunogenicity. They even function as antigen-presenting cells [184], are recognized by T lymphocytes, and induce their proliferation [146]. Therefore, the time lag in the expression of MHC-I and MHC-II observed in MSCs stimulated with IFN-γ, as well as the effect of other cytokines and their possible synergistic effect on the induction of these molecules, must be considered.

It has been reported that IFN-γ gradually increases the expression of MHC-I in MSCs in the first 24 h of treatment, and this effect is maintained for several days [67,107,146,182,183,185]. In contrast, stimulation with TNF-α increases the expression of this molecule between 24 and 48 h after treatment [107]. Even when using high concentrations of this cytokine in BM-MSCs, increased expression of MHC-I has been observed 48 h after stimulation [74,183]. However, the combination of TNF-α and high concentrations of IFN-γ for 48 h exerts a synergistic effect on the expression of MHC-I in BM-MSCs [74,107], which was not observed when the cells were treated with a combination of TNF-α and low concentrations of IFN-γ (5 ng/mL) [107] (Figure 4).

Likewise, TNF-α and IFN-γ induce the expression of MHC-II in MSCs. Regarding the effect of IFN-γ, there is still controversy regarding the exposure times necessary to increase the levels of this molecule. Some studies indicate that treatment with low concentrations of this cytokine for 4 to 8 h is sufficient [184], while others report the need for 24 [132], 48–72 h [74,85,146,148], or more days of exposure [9,146,147]. Importantly, cells stimulated with IFN-γ maintain high expression levels of MHC-I and MHC-II for several days [161]. Interestingly, TNF-α does not affect MHC-II levels in MSCs [36,74,183]. These results highlight the importance of establishing adequate concentrations and exposure times to identify windows in which MSCs maintain a low immunogenic state, a desirable characteristic for them to be used in cell therapy protocols (Figure 4).

## 12. Increased Expression of Immunoregulatory Molecules

Given the importance of cell–cell contact, it is desirable to achieve an increase in the presence of adhesive molecules in the membrane of MSCs during in vitro conditioning protocols. ICAM-1 promotes the migration of MSCs, facilitates their adhesion to immune cells, and is essential for the immunoregulatory function of these cells [140]; thus, its expression should be increased by in vitro conditioning protocols. Resting MSCs express ICAM-1 at low levels [25,107,147,183], while stimulation with IFN-γ significantly increases the percentage of ICAM-1+ cells in the first 24 h after stimulation [107]. Similar results have been observed in AT-MSCs from normal donors [37] and patients with systemic lupus erythematosus, systemic sclerosis, and ankylosing spondylitis [186].

Although IFN-γ is capable of increasing the expression of ICAM-1 and VCAM-1 in BM-MSCs [55,76,147], it has been proposed that TNF-α is the cytokine that provides the initial stimulus to these cells. Recently, TNF-α was shown to increase ICAM-1 levels in human BM-MSCs after 6 h of treatment [107]. In fact, at 2 h, the nuclear translocation of nuclear factor-kappaB (NF-κB) was observed, the activation of which seems to be necessary to induce the immunoregulatory activity of MSCs toward T lymphocytes [138]. Additionally, the presence of tumor necrosis factor receptor 2 (TNFR2) is essential for the adequate expression of immunoregulatory molecules, such as NO, IL-10, and TGF-β, by murine BM-MSCs [187]. This evidence indicates the importance of TNF-α in the activation of MSCs since it favors early expression of an adhesion molecule essential for the immunoregulatory activity of MSCs but not their immunogenic potential.

The importance of IFN-γ in the expression kinetics of other immunoregulatory molecules should be highlighted. In this regard, an increase in the level and activity of the IDO enzyme was observed after 24 h of treatment, which continued to increase at 48 and 72 h, and this effect was maintained for 5 days [10,73,74,157,188]. Similar results have been obtained in the induction of galectin-9 [17,189,190] and PD-L1. In particular, an increase in the expression of PD-L1 was observed after 12 h of exposure to this cytokine, and this trend was maintained at 24, 48, and 72 h [67,74,148] (Figure 4). These results highlight the importance of using both cytokines at appropriate concentrations and times, which allows the attainment of MSCs with a high expression of molecules important for regulating the immune response.

## 13. Effect of Cytokines on EV-MSC Content

Stimulation of MSCs with proinflammatory cytokines increases the amount of EVs released by these cells [117,118,119,120], and changes its content which influences their immunoregulatory capacity. In addition, EVs released by resting or activated BM-MSCs do not express MHC-I or MHC-II [98]. This finding is important because these structures would have a low or no immunogenic capacity.

Human BM-MSCs stimulated with TNF-α and IFN-γ alone or in combination release EVs with a greater ability to interact with immune cells, potentially due to the enrichment of ICAM-1 in exosomes [93] and MVs [107]. The presence of this adhesion molecule would be relevant for the interaction of these structures with their target cells and might facilitate the transfer of immunoregulatory factors [93,98,107,119].

**Figure 5:**
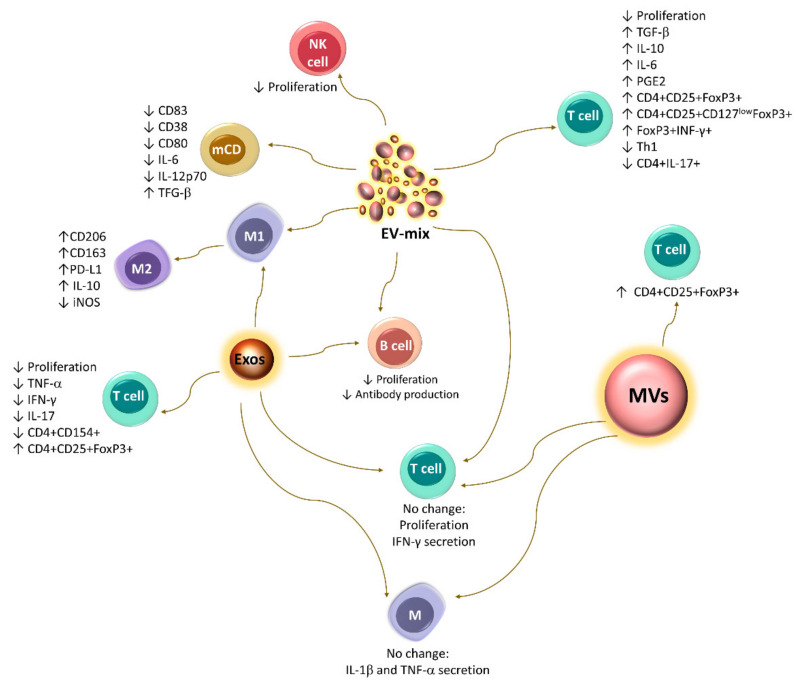
Immunoregulatory effect of EVs released by MSCs. EV-mix preparations decrease the cytotoxic activity of NK cells and the function of mCD, as well as the proliferation of T and B lymphocytes. In addition, they affect the differentiation of Th1 cells while stimulating the differentiation of M2 macrophages and Tregs. However, it has also been reported that this type of preparation does not affect the proliferation and secretion of IFN-γ by T lymphocytes. Similar observations have been made with isolated exosomes (Exos) or MVs and their effect on T lymphocytes and macrophages. Conversely, it has been shown that each of these structures separately favors the differentiation of Tregs; in particular, exosomes decrease the secretion of proinflammatory cytokines by activated T lymphocytes.

Studies that obtained a mixture of exosomes and MVs report that resting human BM-MSCs release an EV-mix capable of decreasing the proliferation of NK cells and B lymphocytes. This effect is more evident when these structures are released by MSCs stimulated with TNF-α and IFN-γ, which is associated with the enrichment of ICAM-1, miRNA-155, and miRNA-146 in these vesicles [98]. In turn, the EV-mix released by resting or IFN-γ-activated UCB-MSCs has the same capacity to decrease the proliferation of T lymphocytes and increase the percentage of CD4+CD25+FoxP3+ cells [85]. In contrast, the EV-mix released by resting or activated human BM-MSCs contains the same levels of PD-L1, does not transport the IDO enzyme, and does not affect the proliferation of T lymphocytes [98] (Figure 4 and Figure 5). However, interestingly, the MV-mix released by MSCs exposed to TNF-α and IFN-γ increases the immunoregulatory capacity of MSCs at rest, stimulating them, although in a less pronounced manner, to induce changes similar to those observed when these cells are activated with proinflammatory cytokines, which translates into an increase in their immunoregulatory effect on T lymphocytes [98].

In contrast to previous reports, more specific studies in which populations of exosomes and MVs are isolated have reported that the exposure of BM-MSCs to TNF-α and IFN-γ induces the release of exosomes enriched in ICAM-1, COX-2, and PGE2 [93], with a greater capacity to interact with monocytes and T lymphocytes at rest or activated with lipopolysaccharide (LPS). In addition, these vesicles decrease the production of TNF-α and IFN-γ by activated primary rat splenocytes, and this effect is associated with the enrichment of COX-2 and PGE2 in exosomes [93]. Similarly, MVs released by human BM-MSCs stimulated with IFN-γ are more efficient at inducing the differentiation of CD4+CD25+FoxP3+ Tregs [109] (Figure 4 and Figure 5).

Furthermore, MVs released by human AT-MSCs stimulated with IFN-γ are enriched in IDO mRNA [37], which can be transferred to the target cell. In this regard, it has been reported that the stimulation of these cells with TNF-α and IFN-γ induces the release of exosomes capable of transferring miRNAs to CD14+ monocytes, which increases the expression of CD163 and polarizes the differentiation of macrophages toward an anti-inflammatory M2 phenotype [108]. In addition, it has been observed that the exosomes released by G-MSCs treated with TNF-α, are enriched in CD73 and participate in the polarization of macrophages towards an M2 phenotype, inducing the expression of CD206 in M1 macrophages. This event is associated with a decrease in periodontal bone loss in a murine model of periodontitis [118]. Likewise, in a Triple-transgenic model of Alzheimer’s disease, the intranasal administration of EV-mix, released by human BM-MSC stimulated with TNF-α and IFN-γ, favors an anti-inflammatory environment. In mice, these structures affect the activation of microglia, reducing the percentage of Iba + and CD68 + cells. While in vitro, they induce an anti-inflammatory phenotype, with a higher expression of IL-10 and a decrease in Il-6 and IL-1β [121] (Figure 4 and Figure 5).

A recent study showed that clinical-grade resting WJ-MSCs release PD-L1+ exosomes, while the stimulation of these cells with low concentrations of IFN-γ induces the production of exosomes enriched in PD-L1 (Exo PD-L1^hi^), which decrease the activation of T lymphocytes, the levels of phosphorylated zeta-chain-associated protein kinase 70 (pZAP70), and the percentage of CD4+CD154+ lymphocytes. In addition, an in vivo model of GVHD showed that PD-L1^hi^ exosomes are taken up by PBMCs [106]. Moreover, an immediate increase in PD-L1+ exosomes was observed in the plasma of patients with acute GVHD who were treated with clinical-grade WJ-MSCs, which decreased as time progressed (1–8 h) [106]. These results indicate that these structures can affect the function of their target cells through PD-L1, which highlights the importance of the mechanisms involving cell–cell contact.

The effects of other cytokines on the content of EVs released by MSCs have also been analyzed. Human UCB-MSCs treated with TGF-β, IFN-γ, or their combination for 72 h generate exosomes with the ability to decrease T lymphocyte proliferation, highlighting a synergistic effect between the two cytokines. This phenomenon is explained by the observation that these exosomes are more efficient at generating Tregs (CD25+FoxP3+), which is associated with higher IFN-γ, IL-10, and IDO contents in these structures [110]. Likewise, the stimulation of MSCs derived from dental pulp (DP-MSCs) with IFN-γ, TNF-α, IL-1β increases the release of EV-mix enriched in PD-L1, IDO, and COX2, which decrease the proliferation of CD4+ and CD8+ T lymphocytes. Moreover, the administration of these structures in a delayed-type hypersensitivity mouse model, improves the integrity of the tissues with a higher presence of M2 cells, and decreases the infiltration of CD45+ cells and M1 macrophages [120]. Similar results have been obtained with EV-mix released by murine BM-MSCs exposed to IL-6, TNF-α, and IL-1β, its administration in a murine model of colitis reduces the clinical-pathological signs of the disease. Reduction in the M1 marker iNOS and increase in the M2 marker CD163 is observed in the colon tissue, while in the intestinal lymph node the Treg/Teffectors ratio increases [117]. Moreover, the MVs obtained from 3D cultures of BM-MSCs exposed to ischemic brain extracts are enriched in immunoregulatory and angiogenic cytokines [111]. These studies show the importance of obtaining a deeper understanding of the effects of different inflammatory scenarios on the content of EVs, which will facilitate the design of adequate in vitro conditioning protocols to obtain functional exosomes or MVs in different pathophysiological contexts.

## 14. Conclusions

The design of in vitro activation protocols that improve the immunoregulatory activity of MSCs has been proposed as a strategy to increase their therapeutic success in clinical trials. Therefore, analyzing the effect of proinflammatory cytokines on the expression of molecules by these cells is relevant. Such analyses will allow the identification of the concentrations, exposure times, and types of cytokines that are convenient for generating MSCs with a high immunoregulatory capacity without increasing their immunogenic potential, altering their morphology, or reducing their viability and proliferation. In this sense, the evidence that positions TNF-α as the first stimulus received by MSCs in an inflammatory context, sensitizing them to the subsequent effect of IFN-γ, is important. This cytokine combination produces a synergistic effect on the expression of immunoregulatory molecules by MSCs, which would allow the use of low concentrations of these cytokines. Determining whether this event enhances immunoregulatory activity requires further study. In addition to the above findings and given the importance of cell–cell contact in the establishment of efficient immunoregulation, the participation of EVs becomes relevant as intermediaries of communication between MSCs and immune cells. Although it has been reported that EVs released by MSCs at rest have immunosuppressive activity, it is important to continue analyzing whether in vitro activation protocols favor the enrichment of these structures with immunoregulatory and adhesive molecules, as well as to evaluate their effect on immune cells; such studies will facilitate the clarification of the contradictory results obtained thus far and understanding of the precise function of exosomes and MVs. Altogether, the reviewed findings will contribute to the establishment of conditioning protocols that make it possible to obtain MSCs or EVs with high immunoregulatory and functional capacity in the different pathophysiological scenarios.

## Figures and Tables

**Table 1:** Isolation method used and type of EVs obtained.

Name Used in the Original Report Cell Source and In Vitro Conditioning Method	Isolation Method	Structures Obtained and Size Range	Study Model	Ref.
**Extracellular vesicles** Human TA-MSCs IFN-γ (50 ng/mL) for 48 h.	Exosome isolation reagent (Invitrogen).	Microvesicles ≈150–500 nm Mean: 262.4 nm	In vitro: T lymphocyte proliferation and differentiation.	[37]
**Extracellular vesicles** Human and murine TA-MSCs Resting MSCs	MVs and Exo preparations: (a) 300× *g* for 10 min; (b) 2000× *g* for 10 min; (c) 0.8 µm membrane filtration; (d) 12,500× *g* for 20 min at room temperature (pure MV isolates); (e) Removing the residual MVs by centrifugation at 20,500× *g* for 40 min; (f) 0.22 µm membrane filtration; (g) 100,000× *g* for 70 min (pure Exo isolates).	MVs: Mean: 400–500 nm Exosomes: Mean: 80–100 nm	In vitro: Proliferation and secretion of cytokines by T lymphocytes.	[48]
**Extracellular** **membrane vesicles** Human UCB-MSCs IFN-γ (100 ng/mL) for 24 and 48 h.	(a) 2000× *g* for 20 min; (b) 100,000× *g* for 1–2 h.	EV-mix 20–700 nm	In vitro: T lymphocyte proliferation and induction of regulatory T cells. In vivo:Ischemia-reperfusion-induced acute kidney injury rat model.	[85]
**Extracellular Vesicles** Human BM-MSCs Resting MSCs	(a) 400× *g* for 5 min; (b) 2000× *g* for 20 min; (c)10,000× *g* for 45 min; (d) 100,000× *g* for 90 min.	EV-mix 152 ± 23 nm	In vitro: Maturation and secretion of cytokines by CDs.	[87]
**Microvesicles** Murine BM-MSCs Resting MSCs	(a) 300× *g* for 10 min; (b) 1000× *g* for 20 min; (c) 10,000× *g* for 30 min; (d) 100,000× *g* for 2 h.	EV-mix 50–200 nm	In vitro: Proliferation and secretion of cytokines by T lymphocytes. Induction of regulatory T cells.	[90]
**Microvesicles** Human WJ-MSCs Resting MSCs	(a) 2000× *g* for 20 min; (b) 100,000× *g* for 1 h.	EV-mix 30–500 nm	In vivo: Ischemia-reperfusion-induced acute kidney injury rat model.	[91]
**Microvesicles** Human BM-MSCs Resting MSCs	(a) 1500× *g* for 20 min; (b) 10,000× *g* for 20 min; (c) 100,000× *g* for 1 h.	EV-mix 60–160 nm	In vitro: Proliferation and secretion of cytokines by T lymphocytes. Induction of regulatory T cells.	[92]
**Extracellular vesicles** Human BM-MSCs TNF-α (20 ng/mL) plus IFN-γ (20 ng/mL) during the night	(a) 0.2-µm membrane filtration; (b) Millipore Lab-scale TFF system equipped with a Biomax 500 kDa Pellicon filter.	Exosomes 75–165 nm	In vitro: Cytokine secretion by activated primary rat splenocytes.	[93]
**Extracellular vesicles** Murine TA-MSCsResting MSCs	(a) 2000× *g* for 20 min; (b) 100,000× *g* for 2 h.	EV-mix 100–1000 nm	In vitro: T lymphocyte proliferation and induction of regulatory T cells.	[94]
**Microvesicles** Human BM-MSCs Resting MSCs	100,000 g for 1 h at 4°C twice.	EV-mix ≈200 nm	In vitro: Activation of murine mast cells.	[95]
**Microvesicles** Human BM-MSCs Resting MSCs	(a) 300× *g* for 20 min; (b) 100,000× *g* for 1 h.	EV-mix 50–200 nm	In vitro: Human type II alveolar cells.	[96]
**Microvesicles** Human BM-MSCs Hypoxia-induced MSCs	a) 1500× *g* for 15 min; b) 0.22 μm membrane filtration; c) 170,000× *g* for 5 h.	Exosomes 50–100 nm	In vitro: Uptake by human umbilical cord endothelial cells.	[97]
**Extracellular vesicles** Human BM-MSCs IFN-γ (10 ng/mL) plus TNF-α (15 ng/mL) for 40 to 48 h.	(a) 300× *g* for 10 min; (b) 2000× *g* for 30 min; (c) 100,000× *g* for 90 min.	EV-mix ≈60–400 nm	In vitro: Proliferation and secretion of cytokines by NK, T, and B cells.	[98]
**Exosomes** Human BM-MSCs Resting MSCs	(a) 300× *g* for 10 min; (b) 10,000× *g* for 20 min; (c) 0.2 µm membrane filtration; (d) 100,000× *g* for 60 min.	Exosomes 65–100 nm	In vitro: Proliferation and differentiation of T and B lymphocytes.	[99]
**Extracellular vesicles** Human BM-MSCs Resting MSCs	(a) 2000× *g* for 20 min; (b) 100,000× *g* for 2 h.	EV-mix 61–121 nm	In vitro: Polarization of macrophages.	[100]
**Exosomes** MSCs from carcinoma and healthy breast tissue Resting MSCs	Exosome Isolation Kit (Invitrogen).	Exosomes Size not reported	In vitro: Polarization of macrophages.	[101]
**Extracellular vesicles** Human BM-MSCs IFN-γ (10 ng/mL) plus TNF-α (15 ng / ml) for 4 h.	(a) 300× *g* for 10 min; (b) 2000× *g* for 30 min; (c) 100,000× *g* for 90 min.	EV-mix ≈25–500 nm	In vitro: Induction of regulatory T cells. In vivo: A xenograft mouse model with steroid-refractory acute graft-versus-host disease.	[102]
**Extracellular vesicles** Human UC-MSCs Resting MSCs	(a) 300× *g* for 10 min; (b) 2000× *g* for 20 min; (c) 10,000× *g* for 30 min; (d) 0.2 µm membrane filtration; (e) 100,000× *g* for 90 min.	Exosomes 105.1–181.1 nm Mean: 139.2 nm	In vivo: Murine model of chronic graft-versus-host disease. Infiltration and activation of macrophages.	[103]
**Extracellular vesicles** Human WJ-MSCs Resting MSCs	a) 10,000× *g* for 20 min; b) 100,000× *g* for 1 h.	EV-mix 164 ± 10.4 nm	In vivo: Murine model of lung ischemia-reperfusion injury. Cytokine expression levels.	[104]
**Extracellular vesicles** Human UC-MSCs Resting MSCs	(a) 2000× *g* for 20 min; (b) 100,000× *g* for 60 min.	EV-mix 80–1000 nm	Single-center, randomized, placebo-controlled, phase II/III clinical pilot study. Patients with grade III-IV chronic kidney disease.	[105]
**Small extracellular vesicle** **Human WJ-MSCs** IFN-γ (2.5 ng/mL) Time is not reported	(a) 400× *g* for 10 min; (b) 2000× *g* for 30 min; (c) 10,000× *g* for 1.5 h; (d) 100,000× *g* for 90 min.	Exosomes 30–150 nm	In vitro: Activation of T lymphocytes. In vivo: Patients with acute graft-versus-host disease.	[106]
**Microvesicles** Human BM-MSCs IFN-γ (10 ng/mL) for 72 h	a) 500× *g* for 15 min; b) 2000× *g* for 20 min; c) 17,000× *g* for 60 min.	Microvesicles 130–1000 nm	In vitro: Analysis of changes in the transport of HLA-I and ICAM-1.	[107]
**Exosomes** Human TA-MSCs IFN-γ plus TNF-α (10, 20 and 40 ng/mL) for 48 h.	ExoQuick-TC System Biosciences.	Exosomes 115 ± 11.5 nm	In vitro: Polarization of macrophages.	[108]
**Microvesicles** Human BM-MSCs IFN-γ (10 ng/mL) for 48 h, 74 h, or 4 days	(a) 300× *g* for 30 min; (b) 16,500× *g* for 20 min.	MVs ≈150 nm	In vitro: Induction of regulatory T cells.	[109]
**Exosomes** UC-MSCs TGF-β (10 ng/mL) IFN-γ (1000 IU/mL) for 72 h. Alone or combined	(a) 3000× *g* for 30 min; (b) 0.22 μm membrane filtration;PEG6000 was added; (c) 3000 rpm for 30 min.	Exosomes 141.6 ± 23.3 nm	In vitro: Induction of regulatory T cells.	[110]
**Microvesicles** Human BM-MSCs Ischemic brain extract for 24 h.	(a) 2500× *g* for 10 min; (b) 14,000× *g* for 45 min at 10 °C.	MVs 150–450 nm	In vitro: Analysis of the content of immunoregulatory molecules.	[111]
**Extracellular vesicles** Murine TA-MSC Resting MSCs	MVs and Exo preparations: (a) 300× *g* for 10 min; (b) 0.8 µm membrane filtration; (c) 12,600× *g* for 30 min (MVs); (d) 0.22 µm membrane filtration; (g) 100,000× *g* for 70 min (Exo).	MVs: Mean: 271 nm Exosome: Mean: 90 nm	In vitro: Mouse-derived peritoneal macrophages.	[112]
**Extracellular Vesicles** Human UC-MSCs Resting MSCs	(a) 3200× *g* for 10 min; (b) 10,000× *g* for 30 min; (c) 100,000× *g* for 2 h.	EV-mix ≈100–300 nm	In vitro: Activation of CD4 + cells. In vivo: Mouse liver ischemia/reperfusion injury model.	[113]
**Smallextracellular vesicles** Human UC-MSCs Resting MSCs	(a) 300× *g* for 10 min; (b) 2000× *g* for 10 min; (c) 10,000× *g* for 30 min; (d) 100,000× *g* for 70 min.	EV-mix 40–200 nm	In vivo: Rat model of rheumatoid arthritis T lymphocyte proliferation, apoptosis, and differentiation.	[114]
**Extracellular Vesicles** Human TA-MSCs Resting MSCs	(a) 0.22 μm membrane filtration; (b) 30,000× *g* for 20 min; (c) 120,000× *g* for 3 h.	Exosomes 90–120 nm	In vivo: Mouse model of dextran sodium sulfate-induced colitis.	[115]
**Extracellular vesicles** Human PL-MSC Resting MSCs	(a) 500× *g* for 10 min; (b) 2000× *g* for 20 min; (c) 5000× *g* for 30 min; (d) 0.2 µm membrane filtration; (e) 130,000× *g* for 2 h.	Exosomes 85–125 nm	In vivo: Mouse model of colitis-induced with trinitrobenzene sulfonic acid.	[116]
**Extracellular vesicles** Murine BM-MSCs IL-6 (20 ng/mL), TNF-α (25 ng/mL) plus IL-1β (25 ng/mL) for 24 h	(a) 1200 rpm for 6 min; (b) 0.22 mm filter; (c) Ami-Con filters Ultra-15, regenerate cellulose 100,000 NMWL; Merck Millipore); (d) 3200× *g* at 4 °C for 15 min.	EV-mix ≈30–300 nm	In vivo: Murine model of sodium dextran. Sulfate-induced colitis. Polarization of intestinal macrophages. Regulatory T cell differentiation.	[117]
**Exosomes** Human G- MSCs TNF-α (100 ng/mL) for 48 h	(a) 300× *g* for 10 min; (b) 3000× *g* for 10 min; (c) 20,000× *g* for 30 min; (d) 120,000× *g* for 70 min; (e) Sucrose gradient; (f) 110,000× *g* for 3 h at 4 °C.	Exosomes Control: 123 ± 3.1 nm TNF-α: 164 ± 7.3 nm	In vitro: Macrophage polarization. In vivo: Ligature-induced periodontitis model in mice.	[118]
**Small extracellular vesicles** human TA-MSCs IFN-γ (10 ng/mL) plus TNF-α (15 ng/mL) for 72 h	(a) 800× *g* for 5 min; (b) 2000× *g* for 10 min; (c) 0.22 mm pore filters; (d) 110,000× *g* for 2 h.	EV-mix ≈80–300 nm	In vitro: CD4 T cell proliferation.	[119]
**Extracellular vesicles** DP-MSCs IFN-γ (50 ng/mL), TNF-α (10 ng/mL) plus IL-1β (10 ng/mL) for 48 h	(a) 2000× *g* for 20 min; (b) 10,000× *g* for 70 min; (c) 0.22 μm membrane filtration; (d) 110,000× *g* for 120 min.	EV-mix ≈100–350 nm	In vitro: T cell proliferation. In vivo: Delayed-type hypersensitivity mouse model.	[120]
**Extracellular vesicles** Human BM-MSCs IFN-γ (25 ng/mL) plus TNF-α (20 ng/mL) for 24 or 48 h	(a) Centrifugation to remove cells and cell debris; (b) 110,000× g for 70 min.	EV-mix ≈100–500 nm	In vitro: Murine primary microglia. In vivo: Triple-transgenic model of Alzheimer’s disease.	[121]

Centrifugations were carried out at 4 °C unless another temperature was indicated. MVs: microvesicles; Exo: exosomes; TA: adipose tissue; BM: bone marrow; UCB: umbilical cord blood; WJ: Warton’s jelly; UC: umbilical cord; G: gingival; DP: dental pulp; MSCs: mesenchymal stem/stromal cells.

## Data Availability

Not applicable.
